# The Influence of Climate Change on Droughts and Floods in the Yangtze River Basin from 2003 to 2020

**DOI:** 10.3390/s22218178

**Published:** 2022-10-25

**Authors:** Lilu Cui, Mingrui He, Zhengbo Zou, Chaolong Yao, Shengping Wang, Jiachun An, Xiaolong Wang

**Affiliations:** 1 School of Architecture and Civil Engineering, Chengdu University, Chengdu 610106, China; 2 Key Laboratory of Earthquake Geodesy, Institute of Seismology, China Earthquake Administration, Wuhan 430071, China; 3 Gavitation and Earth Tide, National Observation and Research Station, Wuhan 430071, China; 4 Institute of Disaster Prevention, Sanhe 065201, China; 5 College of Natural Resources and Environment, South China Agricultural University, Guangzhou 510642, China; 6 College of Geomatics, East China University of Technology, Nanchang 330013, China; 7 Key Laboratory of Marine Environment Exploration Technology and Application, Ministry of Natural Resources, Guangzhou 510030, China; 8 Chinese Antarctic Center of Surveying and Mapping, Wuhan University, Wuhan 430079, China; 9 Nanning Survey and Design Institute Group Co., Ltd., Nanning 530022, China

**Keywords:** hydrological disaster, YRB, GRACE/GRACE-FO, ENSO, IOD

## Abstract

In recent decades, extreme floods and droughts have occurred frequently around the world, which seriously threatens the social and economic development and the safety of people’s lives and properties. Therefore, it is of great scientific significance to discuss the causes and characteristic quantization of extreme floods and droughts. Here, the terrestrial water storage change (TWSC) derived from the Gravity Recovery and Climate Experiment (GRACE) and its Follow-On (GRACE-FO) data was used to characterize the floods and droughts in the Yangtze River basin (YRB) during 2003 and 2020. To reduce the uncertainty of TWSC results, the generalized three-cornered hat and least square methods were used to fuse TWSC results from six GRACE solutions. Then combining precipitation (PPT), evapotranspiration, soil moisture (SM), runoff, and extreme climate index data, the influence of climate change on floods and droughts in the YRB was discussed and analyzed. The results show that the fused method can effectively improve the uncertainty of TWSC results. And seven droughts and seven floods occurred in the upper of YRB (UY) and nine droughts and six floods appeared in the middle and lower of YRB (MLY) during the study period. The correlation between TWSC and PPT (0.33) is the strongest in the UY, and the response time between the two is 1 month, while TWSC and SM (0.67) are strongly correlated with no delay in the MLY. The reason for this difference is mainly due to the large-scale hydropower development in the UY. Floods and droughts in the UY and MLY are more influenced by the El Niño-Southern Oscillation (ENSO) (correlation coefficients are 0.39 and 0.50, respectively) than the Indian Ocean Dipole (IOD) (correlation coefficients are 0.19 and 0.09, respectively). The IOD event is usually accompanied by the ENSO event (the probability is 80%), and the hydrological hazards caused by independent ENSO events are less severe than those caused by these two extreme climate events in the YRB. Our results provide a reference for the study on the formation, development, and recovery mechanism of regional floods and droughts on a global scale.

## 1. Introduction

Droughts and floods are the two most destructive hydrological disasters in the world, which caused huge adverse influences on infrastructure, industrial and agricultural production, and human life safety [1,2]. With the rapid development of human society and population growth, as well as global warming, the frequency and severity of floods and droughts have been increasing in recent decades [3,4]. Therefore, we urgently need to establish an early warning mechanism for floods and droughts to ensure the sustainable development of human society. And it has very important scientific value and social significance that a comprehensive and accurate analysis of the influencing factors affecting floods and droughts.

The Yangtze River basin (YRB) is the largest in China and the third largest in the world. Except for some regions on the Qinghai-Tibet Plateau, other regions are in the monsoon climate zone. The middle and lower reaches (MLY) are mainly affected by the East Asian monsoon, while the upper reaches (UY) are controlled not only by the East Asian monsoon but also by the Indian Ocean summer monsoon [5]. Jiang et al. [6] used historical hydrological data to study the relationship between hydrological disasters and El Niño-Southern Oscillation (ENSO) in the YRB on a time scale of 600 years. The results indicate that ENSO may affect the YRB through the East Asian monsoon. Zhang et al. [7] and Wei et al. [8] studied the relationship between runoff and ENSO in the YRB. The results show that the UY and MLY are affected differently by ENSO. Li et al. [9] and Chen et al. [10] analyzed the characteristics of precipitation (PPT) in the YRB under the combined action of ENSO and Indian Ocean Dipole (IOD). The results show that IOD has a significant influence on the climate of the YRB, especially the UY.

The Gravity Recovery and Climate Experiment (GRACE) and its follow-on (GRACE-FO) missions are jointly developed and managed by the National Aeronautics and Space Administration (NASA) and Deutschen Zentrums für Luft-und Raumfahrt. The GRACE/GRACE-FO provides a new approach to detecting terrestrial water storage change (TWSC) through time-variable gravity signals [11,12]. The TWSC is an important part of the global water cycle, which includes soil moisture (SM), groundwater storage change, runoff and glacier mass change, etc. And it’s closely related to PPT and evapotranspiration (ET), which can be used to quantitatively analyze global or regional climate changes [13,14,15]. Therefore, the use of GRACE/GRACE-FO to detect global or regional TWSC can provide important data support for studying the influence of global climate change on the regional terrestrial water cycle. Zhang et al. [16] analyzed the relationship between droughts and ENSO in the YRB between 2006 and 2011, based on GRACE TWSC data. The results indicate that ENSO has significantly correlated with TWSC, and the lower reaches are more sensitive to ENSO than the UY. Li et al. [17] used GRACE time-variable gravity field data to study the droughts in Southwest China from 2009 to 2010. The results show that the drought was caused by higher temperatures and less PPT. Forootan et al. [18] analyzed the connection between droughts and extreme climate in 156 basins around the world by using GRACE TWSC data. The results show that ENSO has a global influence on droughts, and the most significant regions include most of Asia, Australia, the Amazon River basin, and North Africa. However, IOD and North Atlantic Oscillation only have a regional influence on droughts. Reager et al. [19] evaluated the possibility of using GRACE TWSC and PPT data to predict the occurrence of floods in the Niger River basin. The results indicate that the flood index based on GRACE TWSC and PPT data has good consistency with the local flood data.

However, the above research has the following shortcomings: (1) The influence of the differences in the GRACE solutions from different institutions on the study results was not considered; (2) They mainly focused on the detection and cause analysis of droughts and floods, and did not quantify the characteristics of the flood; (3) Lack of discussion on the influence of extreme climate interaction on regional climate. Based on the above problems, we firstly fused the TWSC results from six GRACE solutions by using the generalized three-cornered hat (GTCH) and least square method to improve the reliability of our results. Then, we characterize the floods and droughts in the YRB, UY, and MLY during 2003 and 2020, respectively. Finally, we applied the cross-correlation analysis method to discuss the influence of natural factors on floods and droughts in the UY and MLY, and compared and analyzed the influence of ENSO and IOD on hydrological disasters in the UY and MLY under the interconnection of these two extreme climates. Quantitative assessment and cause analysis of extreme floods and droughts will help to achieve early warning of disasters and successfully carry out disaster relief work. Our research is of great scientific significance for understanding the triggering mechanism of regional floods and droughts and assessing disaster losses around the world. This paper is organized as follows. We briefly introduced the study area, data, and methods in Section 2 and Section 3, respectively. Section 4 presents the analysis results about the characteristics of hydrological disasters and the influence of climate change on floods and droughts in YRB. The discussion and conclusion are provided in Section 5 and Section 6, respectively.

## 2. Study Area and Data

### 2.1. Study Area

**Figure 1:**
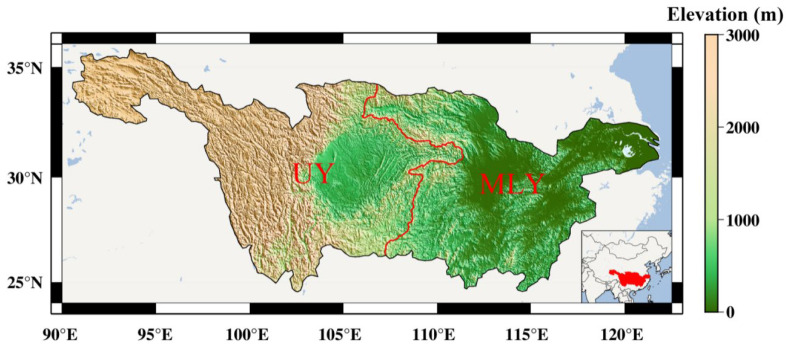
Digital elevation model of YRB (UY: the upper of YRB; MLY: the middle and lower of YRB).

The YRB, approximately at 24° N–36° N and 90° E–121° E, spans the three major economic regions of eastern, central, and western China, with a total of 19 provinces-level administrative regions, and is the third largest drainage basin in the world, with a total basin area of 1.8 million km^2^ (see Figure 1) [20]. In this region, the annual average temperature is high in the east and low in the west, and high in the south and low in the north. The hottest month is July and the coldest one is January. April and October are the middle months of warm and cold changes. The average annual PPT and ET are 1067 mm and 541 mm, respectively [21]. 

There are convenient transportation and abundant water resources in the YRB. The YRB has always been an important industrial and agricultural base in China, whose region’s domestic product is about 40.3 trillion yuan, accounting for 44.1% of China. And it’s also one of the cultural cradles of the Chinese nation.

### 2.2. Data

#### 2.2.1. GRACE/GRACE-FO Data

In our study, we used two types of GRACE/GRACE-FO solutions to obtain the monthly 1° × 1° gridded TWSC data. The above two types of solutions are spherical harmonic (SH) and Mascon solutions, respectively. The SH solutions (truncated to degree and order 60) were provided by the Center for Space Research at the University of Texas at Austin (CSR), Helmholtz-Centre Potsdam-German Research Centre for Geosciences (GFZ), Jet Propulsion Laboratory (JPL) and Institute of Geodesy at Graz University of Technology (ITSG), respectively, while the Mascon solutions were provided by CSR and JPL.

The Mascon solutions can directly obtain the global TWSC gridded data without any data processing. However, the SH solutions need a series of data processing to obtain TWSC gridded data. The processing flow is as follows: firstly, the results of Swenson et al. [22] were used to correct the degree−1 coefficients of SH solutions due to geocentric motion. Secondly, due to satellite orbit, the accuracy of the C_20_ coefficient of the SH solution is low. The C_20_ coefficient is related to the flatting of the Earth. Therefore, the C_20_ coefficient of satellite laser ranging was used to replace it [23]. Thirdly, the de-correlation P3M6 and 300 km Gaussian filter were used to weaken the strip and high-frequency errors of SH coefficients. Finally, due to degree truncation and filtering processing, there is a signal attenuation problem. Therefore, we used the scale factor method [24] to recover the signal. 

In our study, four SH solutions and two Mascon solutions were used to obtain GRACE/GRACE-FO TWSC gridded data from January 2003 to June 2017 and from June 2018 to December 2020. Since GRACE and GRACE-FO data are essentially the same, we referred to GRACE and GRACE-FO data as GRACE data. For convenience, the four SH solutions and two Mascon solutions are termed CSR-SH, GFZ-SH, JPL-SH, ITSG-SH, CSR-M, and JPL-M.

#### 2.2.2. Reconstructed TWSC Data

The reconstructed TWSC dataset in China based on PPT (2002–2019) was used to fill an 11-month data gap between GRACE and GRACE-FO missions, provided by National Tibetan Plateau Data Center. The dataset integrated CSR GRACE/GRACE-FO RL06 Mascon solutions, China’s daily gridded PPT real-time analysis system (version 1.0) and CN05.1 temperature data and other datasets by using the PPT reconstruction model and considering the seasonal items and trend item of CSR RL06 Mascon solutions [25]. The reconstruction expression is as follows [26]:

(1) $$TWSC_{rec}=\beta •P^{\tau }$$

where $TWSC_{rec}$ is the reconstruction TWSC data, P is the monthly PPT data, β is the calibration parameter of the long-term trend term, and τ is the calibration parameter of the seasonal term. 

#### 2.2.3. In Situ PPT Data

We used the in situ PPT gridded data from the China National Meteorological Science Data Center, whose spatial resolution is 0.5° × 0.5°. The dataset was calculated based on the monthly PPT data from all meteorological stations in China since 1961, and the quality of these datasets was checked by using the cross-validation quality test method. In our study, the PPT gridded data is from January 2003 to December 2020. To keep the spatial resolution consistent with GRACE TWSC data, we used the resampling method to process these PPT data to generate 1° × 1° PPT gridded data, and the subsequent ET data also adopted the same processing.

#### 2.2.4. ET Data

The Global Land Evaporation Amsterdam Model (GLEAM) estimated the different components of terrestrial ET separately: transpiration, bared-soil evaporation, interception loss, open-water evaporation, and sublimation. The above datasets were obtained based on observation of surface net radiation, near-surface air temperature, microwave Vegetation Optical Depth and estimates of root-zone soil moisture [27,28]. In our study, the monthly ET gridded data with a spatial resolution of 0.25° × 0.25° is from the GLEAM 3.6a, whose time span is from January 2003 to December 2020.

#### 2.2.5. GLDAS Model

The Global Land Data Assimilation System (GLDAS) model was published and updated jointly by the Goddard Space Flight Center at NASA and the National Center for Environmental Prediction at the National Oceanic and Atmospheric Administration (NOAA). GLDAS used the data from the new generation of ground- and space-based observation systems to constrain the modeled land surface stats [29]. In our study, we used the monthly runoff gridded data with a spatial resolution of 1° × 1° provided by the GLDAS 2.1 Noah model from January 2003 to December 2020.

#### 2.2.6. Extreme Climate Index

ENSO is the wind and sea surface temperature oscillations that occur in the equatorial eastern Pacific, while IOD is caused by the difference in sea surface temperature in different parts of the Indian Ocean. The above two extreme climates have a greater influence on the YRB [16,30], so the floods and droughts in this region need to be considered for ENSO and IOD’s influence. In our study, we used the monthly Niño 3.4 index and the Indian Ocean Dipole Model Index (DMI) [31] from January 2003 to December 2020 to represent the state of ENSO and IOD over time, respectively. The two indices were provided by NOAA.

## 3. Method

### 3.1. Different Datasets Fusion

Due to the discrepancies between different datasets, using only a single dataset for data analysis may lead to unreliable results [32]. Therefore, to minimize the discrepancies and obtain reliable results, the GTCH method was used to estimate the relative uncertainty of six GRACE TWSC data. The use of the GTCH method for uncertain evaluation may not require prior information [33]. The technical detail about the GTCH method can be found in Refs. [34,35].

According to the relative covariance of different observation series by using the GCTH method, the different datasets were fused by taking a weighted average of them [36].

(2) $$X=\sum_{i=1}^{N}p_{i}•X_{i}\left(i=1,2,\cdots ,N\right)$$

where $X_{i}$ and $p_{i}$ are the ith observation time series and its corresponding weight, respectively. N is the number of observations in X. The weights were determined based on estimated variances.

(3) $$p_{i}=\frac{1/r_{ii}}{\sum_{n=1}^{N}1/r_{nn}}$$

where $r_{ii}\left(i=1,2,\cdots ,6\right)$ is the variance of the ith observation series estimated by the GTCH method. The above process was performed grid by grid until we fused the different datasets on all the grid nodes.

### 3.2. The Correlation Coefficient and Delay Months

Suppose the two independent time series are $z_{1}$ and $z_{2}$, τ is the delay factor, the correlation coefficient between $z_{1}$ and $z_{2}$ is expressed as [37]:

(4) $$\rho \left(\tau \right)=\frac{\sigma_{12}\left(\tau \right)}{\sqrt{\sigma_{11}\sigma_{22}}}$$

where ρ(τ) is the correlation coefficient, $\sigma_{11}$ and $\sigma_{22}$ is the variance of $z_{1}$ and $z_{2}$, $\sigma_{12}$ is the covariance of $z_{1}$ and $z_{2}$. When |ρ(τ)| is maximum (|ρ(τ)|≤1), τ is the maximum delay months (|τ|≤12).

## 4. Results

### 4.1. Data Fusion

**Figure 2:**
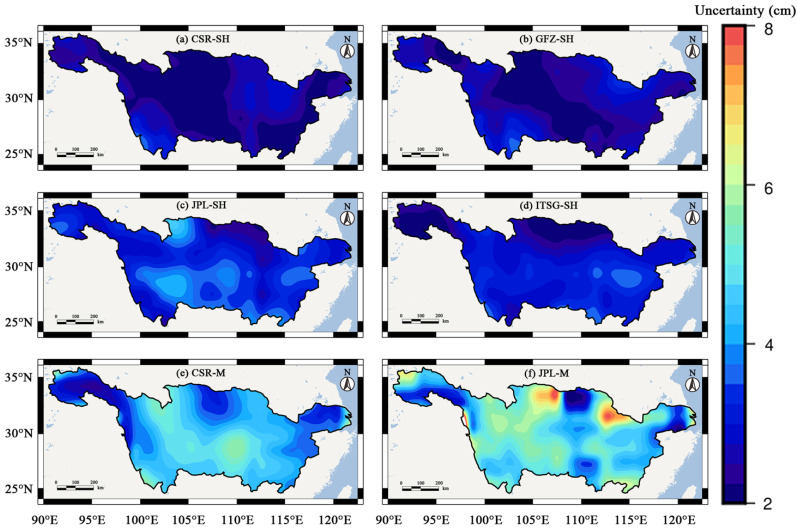
The spatial distribution of uncertainties of TWSC results in the YRB derived from CSR-SH, GFZ-SH, JPL-SH, ITSG-SH, CSR-M, and JPL-M solutions estimated by the GTCH method.

We provided the spatial distribution of uncertainties of TWSC results from six GRACE solutions in the YRB (Figure 2). From Figure 2, the GRACE TWSC results from Mascon solutions exhibit smaller uncertainties (less than 4 cm) than those from SH solutions (from 4 cm to 6 cm in most regions). In Figure 2f, the two regions with greater uncertainties (7~8 cm) appeared in the northern YRB. It suggests that the total uncertainty level of JPL-M is greater than those of the other five solutions.

To compare the total uncertainty level of these six TWSC results, the median of all the grid values of the uncertainty of YRB was used to represent the total uncertainty level of TWSC results from the different GRACE solutions. In ascending order according to the total uncertainty level, the order of these six GRACE solutions is CSR-SH (2.51 cm), GFZ-SH (2.73 cm), ITSG-SH (2.94 cm), JPL-SH (3.22 cm), CSR-M (4.62 cm) and JPL-M (5.20 cm).

**Figure 3:**
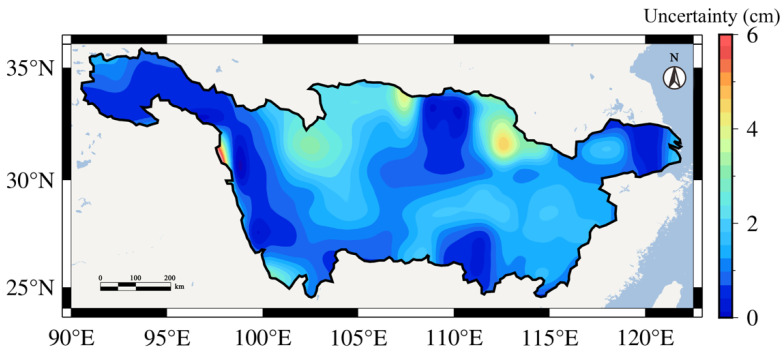
The spatial distribution of uncertainties of fused TWSC results in the YRB.

To improve the reliability of our study results, we fused these six GRACE TWSC results according to the uncertainty results in Figure 2. The spatial distribution of uncertainties of the fused result was shown in Figure 3. In this figure, the uncertainties are smaller than 2 cm in most regions. Only in the northern YRB, there are two regions with uncertainties of 4–6 cm. Combining Figure 2 and Figure 3, the uncertainty of the fused result has been significantly improved. The total uncertainty level of the fused result (1.29 cm) is significantly smaller than those of a single GRACE solution.

**Figure 4:**
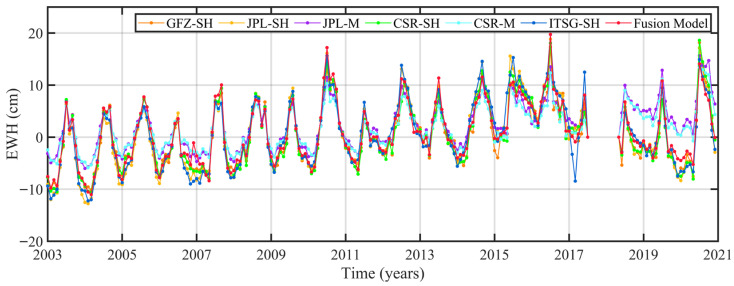
The time series of TWSC derived from six GRACE solutions and fused result in the YRB.

To further evaluate the fused result, we compared the time series of these six GRACE TWSC results and fused results in the YRB (Figure 4). These seven TWSC results have similar trend changes and seasonal variations. The correlation coefficient results show that the fused result has a strong correlation with six GRACE TWSC results (the correlation coefficients are greater than 0.90). Among them, the strongest correlation is CSR-SH (0.9825), followed by GFZ-SH (0.9819), JPL-SH (0.9781), ITSG-SH (0.9697) and CSR-M (0.9184), and finally JPL-M (0.9163). The magnitudes of the fused result are closer to those of SH solutions than the ones of Mascon solutions. It’s due to the greater uncertainties of Mascon solutions. Based on the above analysis, the fused result has a good consistency with six TWSC results from a single solution. Therefore, the fused result was used in the follow-up study.

### 4.2. Droughts and Floods Events in the Study Regions

**Figure 5:**
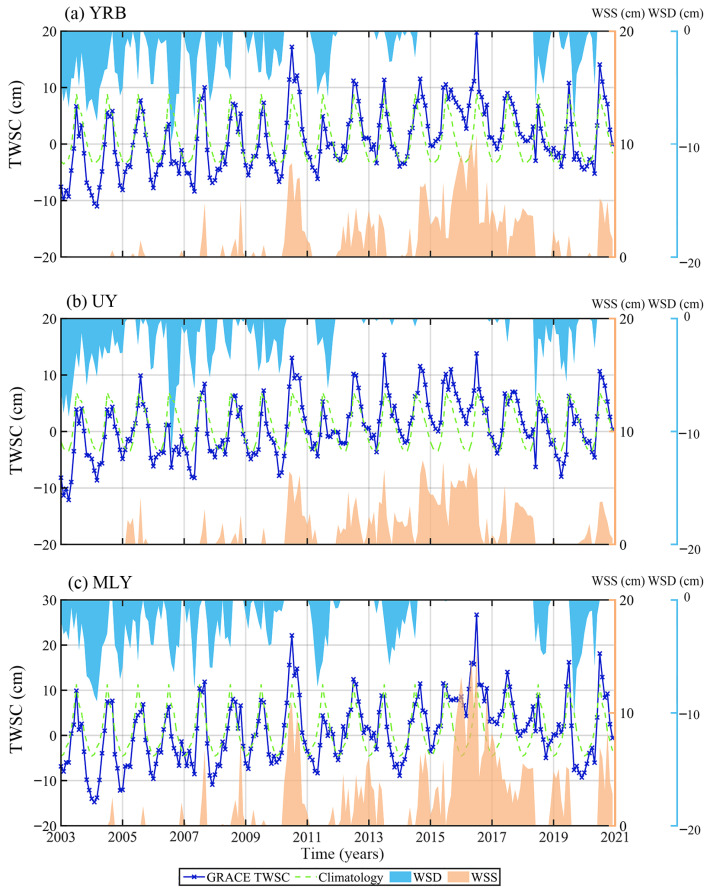
The time series of GRACE TWSC, water storage deficit, and surplus in YRB, UY, and MLY. Navy blue lines: monthly GRACE TWSC, green dotted line: month climatology, light blue shaded regions: water storage deficit, and orange shaded regions: water storage surplus.

To characterize floods and droughts in the YRB, UY, and MLY, we calculated the water storage deficit (WSD) and water storage surplus (WSS) in these regions from 2003 to 2020, respectively (Figure 5). We calculated a 216-month climatology (January 2003 to December 2020) for the GRACE TWSC time series in these regions by averaging each month of GRACE TWSC data. This climatology was used as the baseline for identifying WSD and WSS. WSD and WSS were calculated as the negative and positive residuals after subtracting GRACE climatology from the GRACE TWSC time series. They represent the substantial deviation from the normal annual or seasonal cycle, which was used to distinguish drought and floods [38,39]. WSD corresponds to droughts, while WSS corresponds to floods. From Figure 5, we found that the temporal distribution of WSD (blue-shaded) and WSS (orange shaded) in the three regions is basically the same. WSD mainly appeared in 2003–2012 and 2018–2020, while WSS is mainly concentrated in 2010–2020. The shaded parts can quantify the duration and development of each WSS and WSD, and their areas can represent the WSD and WSS values of each hydrological disaster.

According to the definition of drought events [16], we made statistics on drought events and their characteristics in three regions (Table 1). In this study, we only considered the flood events with WSS lasting three months or more. Therefore, the corresponding statistics results on flood events in three regions are shown in Table 2. We validated the droughts and floods identified by GRACE TWSC data by using the local meteorological droughts and floods data. This local data is from the Ministry of Water Resources of China. From Table 1, there are 8 drought events in the YRB during the study period. The largest drought event occurred from January 2003 to April 2005, which lasted 28 months and the total WSD was −2115.72 km^3^. Seven drought events occurred in the UY from 2003 to 2020. And the largest drought event appeared from January 2003 to February 2005, whose duration and drought severity are 26 months and −1843.74 km^3^. In the same period, there are a total of 9 drought events in the MLY, and the largest drought event lasted from October 2004 to November 2006 for a total of 26 months, whose drought severity is −1854.9 km^3^. Similarly, we can also obtain from Table 2 the number of flood events, the corresponding start and end times, duration, and flood severity in the three regions during the study period.

We found that there are differences in the characteristic of droughts and floods in the UY and MLY. These differences are due to geographical location and topography. The UY is dominated by basins and mountainous regions, located in southwest China and close to the Indian Ocean, while the MLY is dominated by plains, located in southeast China and close to the Pacific Ocean. Therefore, we mainly discussed factors affecting droughts and floods in the UY and MLY in the follow-up study.

### 4.3. The Influence of Natural Factors on Drought and Floods in UY and MLY

**Figure 6:**
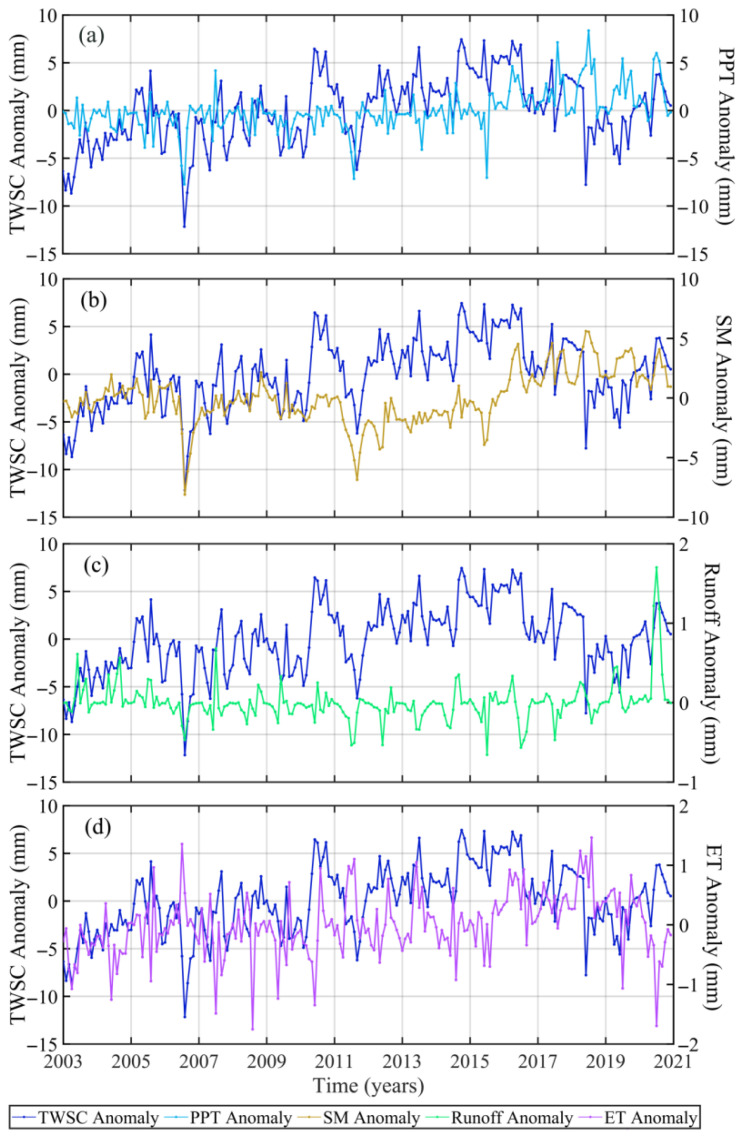
The time series of TWSC and four hydrometeorological factor anomaly ((**a**), PPT; (**b**), SM; (**c**), runoff; (**d**), ET) during 2003~2020 in the UY.

**Figure 7:**
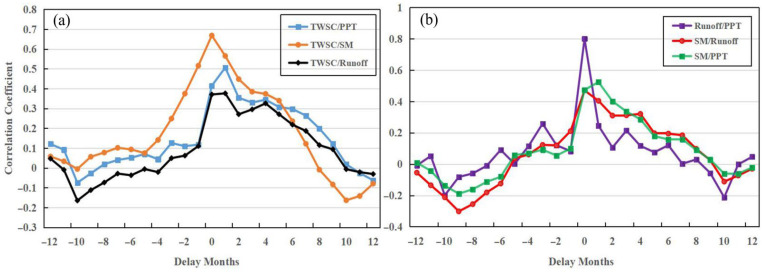
The correlation coefficient and delay months between TWSC, PPT, SM, and runoff anomaly in the UY. (**a**) TWSC and PPT, SM, runoff; (**b**) PPT and runoff, SM, SM and runoff.

**Figure 8:**
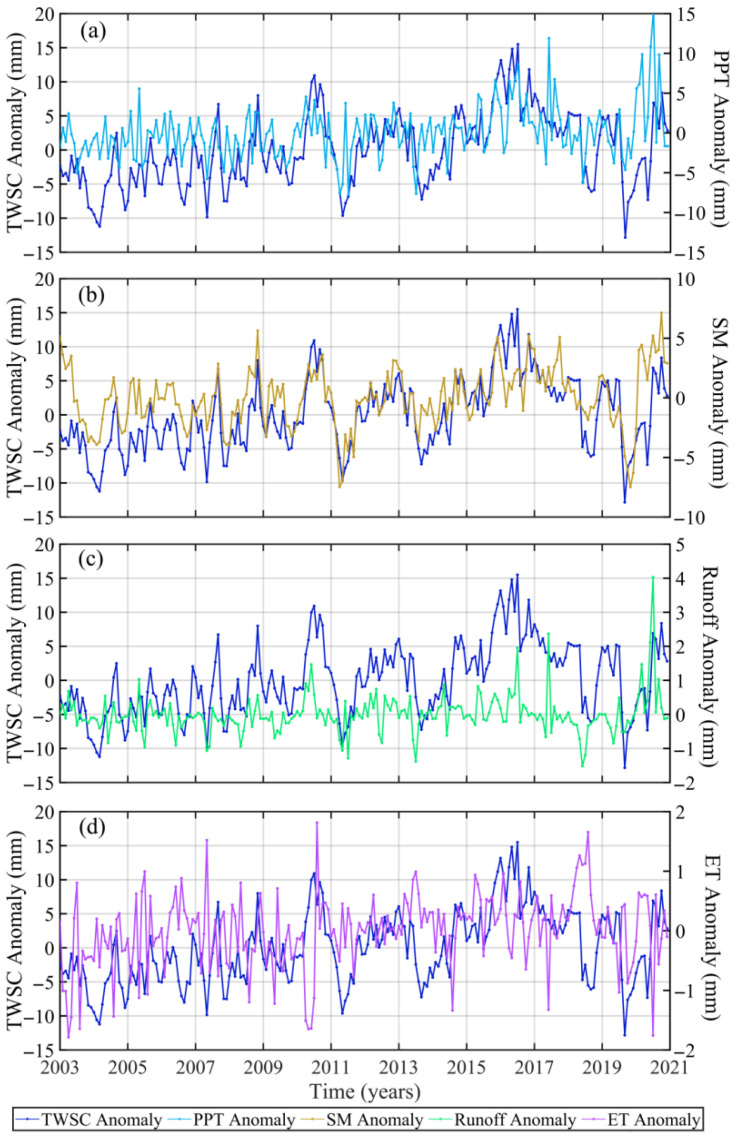
The time series of TWSC and four hydrometeorological factor anomaly ((**a**), PPT; (**b**), SM; (**c**), runoff; (**d**), ET) during 2003~2020 in the MLY.

**Figure 9:**
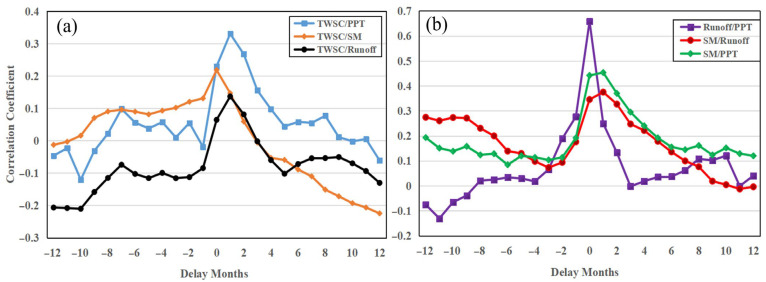
The correlation coefficient and delay months between TWSC, PPT, SM, and runoff anomaly in the LMY. (**a**) TWSC and PPT, SM, runoff; (**b**) PPT and runoff, SM, SM and runoff.

To discuss the influence of climate change on droughts and floods in the UY and MLY, we compared the time series of monthly TWSC, PPT, SM, runoff, and ET anomaly and calculated the correlation coefficients between TWSC anomaly and PPT, SM, ET, and runoff anomaly (Figure 6, Figure 7, Figure 8 and Figure 9, Table 3, Table 4, Table 5 and Table 6). From Figure 6, we found that there is a significant difference between TWSC anomaly and PPT, SM, runoff, and ET anomaly. Table 3 shows the same conclusion, that is, the correlation coefficients between TWSC anomaly and PPT, SM, runoff, and ET anomaly are less than 0.23. It may be due to the continuous hydropower development in the UY in recent decades. Cui et al. [35] indicate that the contribution of reservoir water storage to TWSC in Sichuan (UY) reached 43.32%. And the area of Sichuan accounts for nearly 50% of the UY. Three Gorges Reservoir has a strong correlation (0.55) with the total groundwater storage anomaly in the UY, and groundwater storage is an important component of TWSC [40].

From Table 3, the correlation between TWSC and PPT anomaly is the highest (0.23), followed by SM (0.22), and finally runoff (0.06) and ET (0.06). It explains that droughts and floods are most affected by PPT in the UY, followed by SM, while the influences of runoff and ET are negligible. We also found that PPT is an important source of runoff (the correlation coefficient is 0.66) and has a weaker influence on SM (the correlation coefficient is 0.44). Figure 6 shows that TWSC, PPT, and ET anomaly range from 9 mm to −12 mm, 9 mm to −8 mm, and 1.2 mm to −1.8 mm, respectively. It explains that TWSC anomaly is mainly attributable to PPT anomaly, and ET has little impact. The correlation coefficient results also confirmed this conclusion. Therefore, we focused on the influence of PPT in the follow-up study.

Considering the delay effect, we calculated the maximum correlation coefficients and the delay months between TWSC, PPT, SM, and runoff anomaly (Table 4 and Figure 7a). We found that the response times of TWSC for PPT and runoff are 1 month, while TWSC’s response to SM is not delayed. After the delay, the correlation between TWSC and PPT has improved (0.33), which is higher than that of SM (0.22). It shows that PPT is an important factor affecting droughts and floods in the UY. We also estimated the correlation coefficients and delay months between SM and PPT, runoff and PPT, and SM and runoff (Table 4 and Figure 7b). It states that the response times of SM for PPT and runoff are 1 month, and there is no delay in the response of runoff to PPT. PPT (0.45) has a greater influence on SM than runoff (0.38). It explains that both PPTs have a stronger influence on SM than runoff, and the imbalance of water in the soil is the cause of droughts and floods [15].

Figure 8 shows the time series of TWSC, PPT, SM, ET, and runoff anomaly in the LMY during 2003–2020. The results show that TWSC and SM anomaly have basically the same change trend, which is corroborated by the results of the correlation coefficient (Table 5), that is, TWSC has a strong correlation with SM (0.68). The correlation coefficients between TWSC and PPT, runoff is 0.41 and 0.37, respectively. However, ET has little correlation with TWSC (0.03), which is similar to that in the UY. It suggests that ET has no significant influence on droughts and floods in the YRB. Comparing Table 3 and Table 5, TWSC has a stronger correlation with SM and runoff in the MLY. It is due to the establishment of large-scale hydropower stations in the UY, which form reservoirs of water storage that account for a large proportion of terrestrial water storage [33]. The correlation between TWSC and PPT in the MLY is stronger than that in the UY, which is because the reservoir storage water plays a role in regulating PPT in the UY.

As shown in Table 5, the correlation between PPT, SM, ET, and runoff anomaly in the MLY. A strong correlation only appeared between runoff and PPT (0.80), SM and PPT (0.47). It explains that the rivers absorbed most of the water from PPT. The correlation between runoff and SM is also stronger (0.47), which is the same as that between PPT and SM. It suggests that the recharge of SM by PPT and runoff is not much different. Comparing Table 4 and Table 6, the correlation between PPT and runoff in the MLY (0.80) is stronger than that in the UY (0.66), while there are similar correlations between PPT and SM in the UY (0.44) and MLY (0.47). It shows that the reservoirs in the UY have intercepted part of PPT that should have been in the river basin [41].

We also calculated the maximum correlation coefficients and delay months between TWSC, PPT, SM, and runoff anomaly (Table 6 and Figure 9). Table 6 shows that TWSC has a strong correlation with SM (0.67) and PPT (0.51), while the correlation coefficient between TWSC and runoff is 0.38. It reveals that PPT and SM are major contributors to droughts and floods, which is quite different from the results in the UY. It also explains that human factors have a very important impact on TWSC in the UY. This difference is mainly due to human factors. According to statistics, the water storage capacity of reservoirs in Sichuan Province (UY) has increased sharply, from 12.241 billion cubic meters in 2009 to 52.908 billion cubic meters in 2020, an increase of nearly 5 times [35]. Therefore, the main factor that affects floods and droughts in the UY and MLY is PPT. Combing Table 4 and Table 6, without considering the reservoir water storage, most of the water brought by PPT enters into the river, and small parts go into the soil in the YRB, and the recharge of SM by runoff is weaker than that of PPT.

### 4.4. The Influence of Extreme Climate on Drought and Floods in the UY and MLY

**Figure 10:**
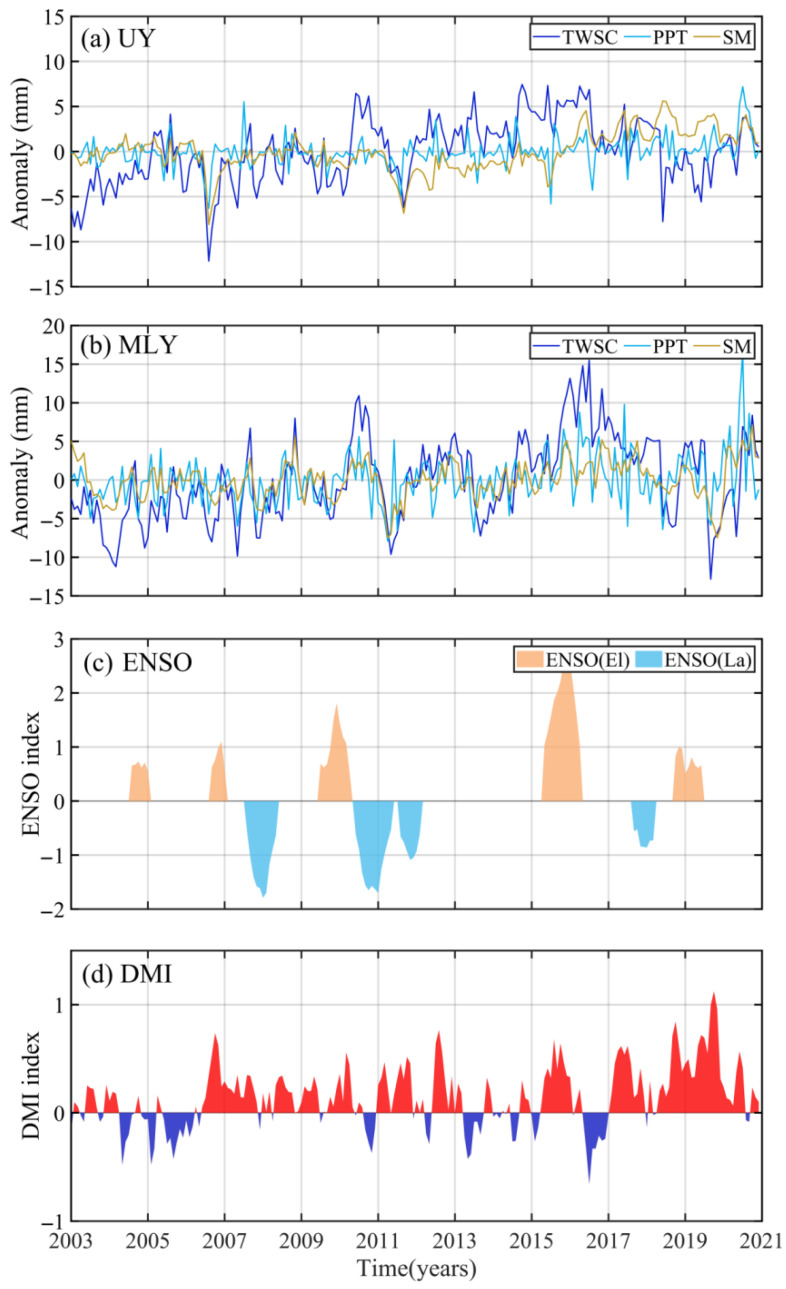
The time series of TWSC, PPT, ET, SM, and runoff anomaly in (**a**) the UY and (**b**) the MLY, (**c**) The temporal distribution of ENSO events, and (**d**) The time series of DMI during 2003~2020. In (**c**), orange shade: El Niño; blue shade: La Niña.

To discuss the influence of extreme climate in the UY and MLY, we compared the time series of TWSC, PPT, and SM anomalies during the ENSO and IOD events (Figure 10). From Figure 10, TWSC has a significant response to ENSO events in the MLY, that is, there is an abnormal increase in TWSC during El Niño events (positive ENSO index, 2004, 2006, 2009, 2015 and 2018), while less TWSC has appeared during La Niña events (negative ENSO index, 2007, 2010, 2011 and 2017). However, there is a certain delay in TWSC’s response to ENSO, about 8 months. We counted the floods and droughts in the YRB during and after ENSO events (Table 7). Except for the 2006 El Niño event, the floods occurred during or after other El Niño events, and the droughts appeared during or after La Niña events. It indicates that the ENSO event has an important influence on the floods and droughts in the YRB, especially in the MLY.

Expect for ENSO, IOD also has a certain influence on the climate of YRB, which can be measured by DMI. When DMI is greater than 0, it corresponds to a positive IOD event, and when DMI is less than 0, it corresponds to a negative IOD event [5]. Figure 10d shows IOD events from 2003 to 2020 (red indicates a positive event and blue indicates a negative event). However, the connection between TWSC anomaly and IOD events is not clear in Figure 10a,b,d. Therefore, we counted the occurrence of floods and droughts during IOD events (Table 8). Here we only focused on the IOD events lasting more than five months. From Table 8, there are nine positive and five negative IOD events. The statistical results show a 77.78% chance of floods during positive events and a 60.00% chance of droughts during negative events. It explains that floods are more likely to occur during positive events than droughts, while the opposite is true during negative events. Table 7 and Table 8 show the influence of ENSO events on extreme disasters in the YRB compared to IOD events.

To further study the influence of ENSO and IOD on the YRB, we calculated the maximum correlation coefficients and delay months between ENSO, DMI and TWSC, PPT, and SM anomaly in the UY and MLY (Table 9). The results show that ENSO has a strong correlation with TWSC and PPT (0.50 and 0.68) in the MLY, and the delay months between ENSO and PPT (5 months) is shorter than that between ENSO and TWSC (8 months). It shows that ENSO has a very important influence on floods and droughts in the MLY, and ENSO acts on PPT to cause floods and droughts, that is, less PPT lead to droughts and more PPT leads to floods, and PPT and TWSC have a certain delay to ENSO. Liu et al. and Zhang [42,43] indicated that less PPT appeared in the MLY during the La Niña event due to the northward shift of the PPT belt, while the situation was opposite during the El Niño event. In the UY, the maximum correlation coefficients between ENSO and TWSC, PPT are only 0.39 and 0.31, respectively. It suggests that the climate changes in the UY and MLY affected by ENSO are different. It is because ENSO mainly affects YRB through the monsoon, and the monsoons affecting different sub-basins are not the same [7,8]. Jin [5] pointed out that the influence of ENSO on the YRB has characteristics from east to west, and its intensity gradually decreases from east to west. And we found that the response time of PPT in the UY to ENSO is also one month longer than that in the MLY. It is due to the geographical location of UY (inland). The correlations between ENSO and SM in the UY and MLY are the same, and the response time of UY is two months longer than that of MLY.

From Table 9, the correlation coefficients between TWSC, PPT, SM, and DMI in the UY are 0.19, 0.49, and 0.27, respectively, which are greater than those in the MLY (0.09, 0.28, and 0.10). Based on the results between TWSC and DMI, the influence of IOD events on floods and droughts in the UY is greater than that in the MLY. The delay months between DMI and PPT are 5 months in UY and MLY, which is shorter than those between DMI and TWSC (6 months). It considers that the positive event led to more PPT in the YRB, while the negative event does the opposite. The conclusion is also consistent with previous studies [44,45]. Li et al. [46] and Anil et al. [47] pointed out that the positive IOD event leads to the strengthening of the Indian Ocean Monsoon and weakening of the South Asian High and Subtropical High, which is favorable for the Indian Ocean Monsoon to transport more water vapor to the UY, while the negative IOD event does just the opposite. The IOD event has a certain influence on PPT in South China, especially Southwest China (UY) [48,49]. And the IOD event also has a certain influence on SM in the UY (0.27), while its influence on SM in the MLY is weaker (0.10).

## 5. Discussion

Table 7 and Table 8 demonstrate that the occurrence of the El Niño event was usually accompanied by the appearance of a positive IOD event. From 2003 to 2020, a total of five El Niño events occurred. During four El Niño events (6 September to 7 January, 9 July to 10 April, 15 April to 16 April, and 18 October to 19 June), the positive IOD events appeared (6 June to 7 November, 9 September to 10 May, 15 April to 16 January and 18 May to 20 July). Luo et al. [50] and Hameed et al. [51] indicated that there is a certain interaction between IOD and ENSO. ENSO affects IOD mainly through the coupled Walker Circulation anomalies between the tropical Indian Ocean and Pacific, and oceanic pathways [52]. And the IOD mainly affects the development of ENSO during the same period by Walker Circulation anomaly and the oceanic fluctuation process related to the zonal wind anomaly [53,54]. Combining Table 2, Table 7 and Table 8, four El Niño events caused the floods. Among them, the El Niño event (4 August to 5 January) is an independent event and the other three El Niño events (9 July to 10 April, 15 April to 16 April, and 18 October to 19 June) were accompanied by the appearance of positive IOD events. The flood in the UY caused by an independent El Niño event is less severe than those by the other three El Niño events, and the total WSS of the above four floods are 37.62 km^3^ (5 March to 5 May), 61.56 km^3^ (10 May to 11 April), 59.28 km^3^ (12 January to 16 September) and 38.24 km^3^ (20 January to 20 December), respectively. In the MLY, in addition to the independent El Niño event, flooding occurred during the other three El Niño events. The above results support the opinion of Annamalai et al. [55], that is, the El Niño event with a positive IOD event is stronger than the independent El Niño event. Overall, both ENSO and IOD have important influences on the climate of YRB, and both often appear at the same time. The IOD event has a strong effect on the influence of the ENSO event. However, due to the complex interaction between ENSO and IOD and their effects on PPT, a large number of studies are still needed.

PPT is the largest contributor to floods and droughts in the YRB, the human activities have a great influence on floods and droughts in the region with frequent human activity (UY). Among many human activities, reservoir water storage has the greatest influence on TWSC in the YRB, especially in the UY. Take Sichuan Province, the largest province in the UY, as an example, the contribution of reservoir water storage to TWSC is as high as 42.32% [35]. Although reservoir water storage can prevent floods and droughts, it also brings serious adverse effects to the regional TWSC. Li et al. [56] suggested that the impoundment of the Three Gorges Dam slightly alleviated the drought in the UY, but significantly exacerbated the drought in the MLY. However, due to some objective factors and the limitation of one’s own ability, it’s difficult to obtain the long-term observation data of reservoir water storage change, and the influence of human activities on floods and droughts was not discussed in our study.

## 6. Conclusions

In this study, the GTCH and least square methods were used to evaluate the uncertainties of six GRACE TWSC results and fuse these TWSC results to improve the accuracy. And we accurately quantified the characteristics of floods and droughts in the YRB and its sub-basins during 2003 and 2020 and analyzed the influence of climate change on these floods and droughts, especially under the combined effect of ENSO and IOD. The results show that the uncertainty of fused TWSC results has been greatly improved, which is beneficial to our study. There are eight droughts and five floods in the YRB between 2003 and 2020. Among them, the most severe flood occurred from August 2014 to May 2018, with a total WSS of 3778.38 km^2^, while the most severe drought appeared between January 2003 and April 2005, with a total WSD of 2115.72 km^2^. The abnormal PPT is an important factor leading to floods and droughts in the YRB, and the extreme climate (ENSO and IOD) is the main black hand causing the abnormal PPT. ENSO and IOD usually occurred together, and IOD has an intensifying effect on ENSO, which results in more severe PPT anomalies than the ones during a single ENSO event in the YRB.

Our results demonstrate the connection between climate change and floods and droughts and the characteristic of hydrological disasters in the YRB and sub-basins. It has very important scientific and practical significance for establishing an early warning mechanism for regional floods and droughts and reducing disaster loss, and also provides a certain reference for global and regional floods and droughts research.

## Figures and Tables

**Table 1:** Summary Table of drought events.

Region	No. of Events	Time Span	Duration (Months)	Average WSD (km^3^)	Total WSD (km^3^)	Local Meteorological Data Validation (Y/N)
YRB 1,800,000 km^2^	8	Jan-03 to Apr-05	28	-75.60	−2115.72	N
		Oct-05 to Jul 07	22	−58.68	−1290.42	Y
		Oct-07 to Jul 08	10	−56.34	−563.76	Y
		Jan-09 to Mar-10	15	−40.32	−604.08	Y
		Mar-11 to Dec-11	10	−53.64	−536.58	Y
		Aug-13 to Jan-14	6	−25.38	−152.64	Y
		Jun-18 to Dec-18	7	−54.18	−379.26	Y
		Apr-19 to Jun-20	15	−37.44	−562.14	Y
UY 1,000,000 km^2^	7	Jan-03 to Fre-05	26	−70.92	−1843.74	N
		Nov-05 to Jul 07	21	−61.2	−1286.82	Y
		Oct-07 to Jan-08	4	−67.32	−269.10	Y
		May-08 to Jul-08	3	−52.02	−155.88	N
		Feb-09 to Apr-10	15	−45.18	−677.52	Y
		May-11 to Nov-11	7	−55.26	−387.36	Y
		Jun-18 to Oct-19	17	−44.82	−763.20	Y
MLY 800,000 km^2^	9	Jan-03 to Jul-04	19	−96.84	−1840.68	N
		Oct-04 to Nov-06	26	−71.28	−1854.90	Y
		Feb-07 to Jul-07	6	−68.94	−413.10	Y
		Oct-07 to Jul-08	10	−75.42	−754.38	Y
		Jan-09 to Mar-10	15	−36.36	−546.48	Y
		Feb-11 to Sep-11	8	−97.02	−776.70	Y
		Jul-13 to Apr-14	10	−69.84	−697.86	Y
		Jun-18 to Nov-18	6	−76.14	−456.48	Y
		Aug-19 to Jun-20	11	−90.36	−993.24	Y

**Table 2:** Summary Table of flood events.

Region	No. of Events	Time Span	Duration (months)	Average WSS (km^3^)	Total WSS (km^3^)	Local Meteorological Data Validation (Y/N)
YRB 1,800,000 km^2^	5	Apr-10 to Feb-11	11	78.12	860.40	Y
		Jan-12 to Jul-13	19	41.58	790.02	Y
		Aug-14 to May-18	46	82.08	3778.38	Y
		Jan-19 to Mar-19	3	30.42	91.08	Y
		Jul-20 to Dec-20	6	64.80	388.98	Y
UY 1,000,000 km^2^	7	Mar-05 to May-05	3	37.62	112.68	N
		Feb-08 to Apr-08	3	17.46	52.20	N
		May-10 to Apr-11	12	61.56	738.90	Y
		Jan-12 to Sep-16	57	59.58	3393.90	Y
		Apr-17 to May-18	14	44.46	621.90	Y
		Nov-19 to Mar-20	5	13.68	68.22	N
		Jun-20 to Dec-20	7	38.34	268.20	Y
MLY 800,000 km^2^	6	Aug-08 to Dec-08	5	47.52	237.96	N
		Apr-10 to Jan-11	10	107.28	1073.16	Y
		Feb-12 to Mar-13	14	53.10	742.86	Y
		Aug-14 to May-18	46	105.12	4836.42	Y
		Dec-18 to Jul-19	8	66.24	529.20	Y
		Jul-20 to Dec-20	6	93.60	561.96	Y

**Table 3:** The correlation coefficients between TWSC, PPT, SM, ET, and runoff anomaly in the UY (these results have passed the 95% confidence level).

Hydrological Component	TWSC	PPT	SM	Runoff	ET
TWSC	1	0.23	0.22	0.06	0.06
PPT	0.23	1	0.44	0.66	−0.26
SM	0.22	0.44	1	0.35	0.16
Runoff	0.06	0.66	0.35	1	−0.28
ET	0.06	−0.26	0.16	−0.28	1

**Table 4:** The correlation coefficients and time delay months between TWSC, PPT, SM, and runoff anomaly in the UY (these results have passed the 95% confidence level).

Components	Correlation Coefficient	Time Delay Months
TWSC/PPT	0.33	1
TWSC/Runoff	0.14	1
TWSC/SM	0.22	0
SM/PPT	0.45	1
Runoff/PPT	0.66	0
SM/Runoff	0.38	1

**Table 5:** The correlation coefficients between PPT, SM, ET, and runoff anomaly in the MLY (these results have passed the 95% confidence level).

Hydrological Component	TWSC	PPT	SM	Runoff	ET
TWSC	1	0.41	0.67	0.37	0.03
PPT	0.41	1	0.47	0.80	−0.33
SM	0.68	0.47	1	0.47	−0.03
Runoff	0.37	0.80	0.47	1	−0.40
ET	0.03	−0.33	−0.03	−0.40	1

**Table 6:** The maximum correlation coefficients and the corresponding time delay months between TWSC, PPT, SM, and runoff anomaly in the MLY (these results have passed the 95% confidence level).

Components	Correlation Coefficient	Delay Months
TWSC/PPT	0.51	1
TWSC/runoff	0.38	1
TWSC/SM	0.67	0
SM/PPT	0.52	1
Runoff/PPT	0.80	0
SM/Runoff	0.47	0

**Table 7:** The drought and flood events statistics during and after ENSO events in the YRB.

ENSO Events		Disaster		
Type	Time Span	Y/N	Type	Location
El Niño	Aug-04 to Jan-05	Y	Floods	UY
El Niño	Sep-06 to Jan-07	N	-	-
La Niña	Aug-07 to May-08	Y	Droughts	UY, MLY
El Niño	Jul-09 to Apr-10	Y	Floods	UY, MLY
La Niña	Jun-10 to May-11	Y	Droughts	MLY
La Niña	Aug-11 to Fre-12	Y	Droughts	UY, MLY
El Niño	Apr-15 to Apr-16	Y	Floods	UY, MLY
La Niña	Sep-17 to Mar-18	Y	Droughts	UY, MLY
El Niño	Oct-18 to Jun-19	Y	Floods	MLY

**Table 8:** The drought and flood events statistics during and after IOD events in the YRB.

IOD Events		Disaster		
Type	Time Span	Y/N	Type	Location
Negative	Apr-04 to Aug-04	Y	Droughts	UY, MLY
Negative	Nov-04 to Mar-05	Y	Droughts	UY, MLY
Negative	Jun-05 to Mar-06	Y	Droughts	UY, MLY
Positive	Jun-06 to Nov-07	Y	Floods	UY, MLY
Positive	May-08 to Oct-08	Y	Floods	UY, MLY
Positive	Dec-08 to Jun-09	Y	Droughts	MLY
Positive	Sep-09 to May-10	Y	Floods	UY, MLY
Positive	Jun-11 to Nov-11	Y	Droughts	UY, MLY
Positive	Jun-12 to Mar-13	Y	Floods	UY, MLY
Negative	Apr-13 to Oct-13	Y	Floods	UY
Positive	Apr-15 to Jan-16	Y	Floods	UY, MLY
Negative	May-16 to Jan-17	Y	Floods	UY, MLY
Positive	Feb-17 to Dec-17	Y	Floods	UY, MLY
Positive	May-18 to Jul-20	Y	Floods	UY, MLY

**Table 9:** The maximum correlation coefficients and the corresponding time delay months between the ENSO index, DMI, and TWSC, PPT anomaly (these results have passed the 95% confidence level).

Variables	Correlation Coefficients		Delay Months	
	UY	MLY	UY	MLY
ENSO vs. TWSC	0.39	0.50	8	8
ENSO vs. PPT	0.31	0.68	6	5
ENSO vs. SM	0.42	0.42	11	9
DMI vs. TWSC	0.19	0.09	6	6
DMI vs. PPT	0.49	0.28	5	5
DMI vs. SM	0.27	0.10	11	8

## Data Availability

GRACE RL06 data from CSR, GFZ, JPL, and ITSG: http://icgem.gfz-potsdam.de/series (accessed on 30 August 2022); In situ precipitation data: http://data.cma.cn (accessed on 30 August 2022); ET gridded data: sftp://hydras.ugent.be (accessed on 30 August 2022); Reconstructed TWSC data: http://data.tpdc.ac.cn (accessed on 30 August 2022); Extreme climate index data: https://psl.noaa.gov/ (accessed on 30 August 2022).
